# Active particles with desired orientation flowing through a bottleneck

**DOI:** 10.1038/s41598-018-27478-y

**Published:** 2018-06-14

**Authors:** Daniel R. Parisi, Raúl Cruz Hidalgo, Iker Zuriguel

**Affiliations:** 1 Instituto Tecnológico de Buenos Aires, CONICET, Lavardén 315, 1437 C A. de Buenos Aires, Argentina; 2 Departamento de Física, Facultad de Ciencias, Universidad de Navarra, E-31080 Pamplona, Spain

## Abstract

We report extensive numerical simulations of the flow of anisotropic self-propelled particles through a constriction. In particular, we explore the role of the particles’ desired orientation with respect to the moving direction on the system flowability. We observe that when particles propel along the direction of their long axis (longitudinal orientation) the flow-rate notably reduces compared with the case of propulsion along the short axis (transversal orientation). And this is so even when the effective section (measured as the number of particles that are necessary to span the whole outlet) is larger for the case of longitudinal propulsion. This counterintuitive result is explained in terms of the formation of clogging structures at the outlet, which are revealed to have higher stability when the particles align along the long axis. This generic result might be applied to many different systems flowing through bottlenecks such as microbial populations or different kind of cells. Indeed, it has already a straightforward connection with recent results of pedestrian (which self-propel transversally oriented) and mice or sheep (which self-propel longitudinally oriented).

## Introduction

In some circumstances, the flow of an ensemble of discrete macroscopic particles through a constriction might display intermittencies. These have been generically characterized by an exponential tail of the flowing intervals distribution and a broad tail (some times a power law) of the clogging times distributions^1^. Such statistical features agree with a description of the intermittent flow in which the probability of blockage formation is constant over time, whereas the probability of getting the block destroyed depends on time. Interestingly, this behavior seems to hold for systems built of different constituents (such as colloids^2–4^, droplets^5^, granular^6–10^, vibration-driven vehicles^11^, animals^12,13^ and pedestrians^14^) as well as for different confining geometries (bottlenecks and obstacle arrays or porous materials^15,16^).

Curiously, the broad tails in the clogging times distributions are not easily reproduced by means of molecular dynamics simulations of discs or spheres, which have evidenced suspicious features such as the apparition of cut-offs^17^ or the necessity of simulating (unphysical) exceedingly long times^1^. Recently, we postulated that introducing an anisotropy in the particles’ shape gave immediately rise to the emergence of robust power law decays of the clogging time distributions^18^. Indeed, the shape of particles has been demonstrated to have a strong impact on the flow^19–23^ and clogging patterns of inert grains when discharged from a silo^24,25^. Also, particle elongation has evidenced to cause the emergence of new features for the case of active particles^26,27^ (see^28^ for a review on this field).

Surprisingly, the number of works where the effect of particle shape is studied for active particles passing through a constriction is scarce. Some of these simulations are within the field of pedestrian dynamics^29–31^ but, for different reasons, contacts among the particles were not implemented; therefore the shape role is expected to be less determinant than in a highly packed situation where particles align each other determining the system response. The only exception to this trend is the work of Alonso-Marroquín *et al*.^32^ who introduced a novel two dimensional section of a human body to simulate counter flow of dense crowds through narrow corridors. Inspired by this paper, we recently simulated the egress of pedestrians by using spherocylinders that aim to propel transversally; i.e. with their longer axis perpendicular to the trajectory^18^. Beyond the undeniable role of the particle shape, both the strength of the torque inducing the desired orientation of the individuals and the magnitude of the noise imposed to erase the blocks at the outlet, were proved to strongly influence the flow rate.

In this manuscript we generalize the results of that previous work and enlarge its scope beyond the field of pedestrian dynamics. To this end, we investigate the role of active desired orientation in the flow of particles through bottlenecks, an issue that -to our knowledge- has been never addressed in the field of active matter. This analysis allows us to discover that the flow dramatically drops when the anisotropic particles propel with the long axis in the displacement direction and suggests that, in order to minimize clogging at confined geometries, a suitable design of artificial active particles might involve self-propulsion without any preferred orientation.

## The Model

The contact force model that allows to simulate highly packed ensembles of spherocylinders was already presented in^18^. Therefore, we will only describe here its main characteristics.

For particle *i*, the equations of motion for the translation of the center of mass and the rotation around it are given by Eqs 1 and 2 respectively,

1 $${m}_{i}{\overrightarrow{\ddot{r}}}_{i}={\overrightarrow{F}}_{Di}+{\overrightarrow{F}}_{Gi},$$

2 $${I}_{i}{\overrightarrow{\ddot{\theta }}}_{i}={\overrightarrow{\tau }}_{Di}+{\overrightarrow{\tau }}_{Gi}$$

where *m*_*i*_ is the particle’s mass, *I*_*i*_ its moment of inertia, ${\overrightarrow{\ddot{r}}}_{i}$ is the linear acceleration and ${\overrightarrow{\ddot{\theta }}}_{i}$ the angular acceleration. In both equations, the first term of the right hand side accounts for the self-propulsion (or energy input provided by the own particle), and the second term for the contact interactions given by walls and neighboring particles.

The contact forces ${\overrightarrow{F}}_{Gi}$ acting on the center of mass of a given particle can be written as

3 $${\overrightarrow{F}}_{Gi}=\sum _{j}^{{N}_{c}}{\overrightarrow{F}}_{ij}+{\overrightarrow{F}}_{wi},$$

where ${\overrightarrow{F}}_{wi}$ is the force of the particle with the wall, and the sum accounts for the forces ${\overrightarrow{F}}_{ij}$ exerted by the *N*_*c*_ contacting neighbors *j* on *i*. These contact forces also produce a torque on the particle *i* that is given by

4 $${\overrightarrow{\tau }}_{Gi}=\sum _{j}^{{N}_{c}}{\overrightarrow{r}}_{ij}\times {\overrightarrow{F}}_{ij}$$

where ${\overrightarrow{r}}_{ij}$ is a vector pointing from the center of particle *i* to the contact point with particle *j*. More details about the numerical implementation of contacts among two spherocylinders can be found in^18^.

Focusing now on the self-propulsion terms of Eqs 1 and 2, ${\overrightarrow{F}}_{Di}$ is the translation-driving force given by:

5 $${\overrightarrow{F}}_{Di}={m}_{i}\frac{({\overrightarrow{v}}_{i}-{\overrightarrow{v}}_{d})}{{\rm{\lambda }}}$$

where ${\overrightarrow{v}}_{i}$ and ${\overrightarrow{v}}_{d}$ = *v*_*d*_**e**_*i*_ are the actual and desired particle velocities respectively. The normalized vector **e**_*i*_ points to the desired target; i.e. towards the exit. Finally, *λ* = 0.5 s is the characteristic time that a free particle (with no contacts) would need to achieve *v*_*d*_.

Implementing anisotropic particles provides the opportunity of introducing a *desired torque*, *τ*_*Di*_, which accounts for the strength with which particles aim to align in a certain orientation. In our case, we define it as:

6 $${\overrightarrow{\tau }}_{Di}=[\,-\,{S}_{D}{\rm{\Delta }}\theta -\beta \dot{\theta }+{\Re }(t\mathrm{)]\ }\hat{z},$$

**Figure 1:**
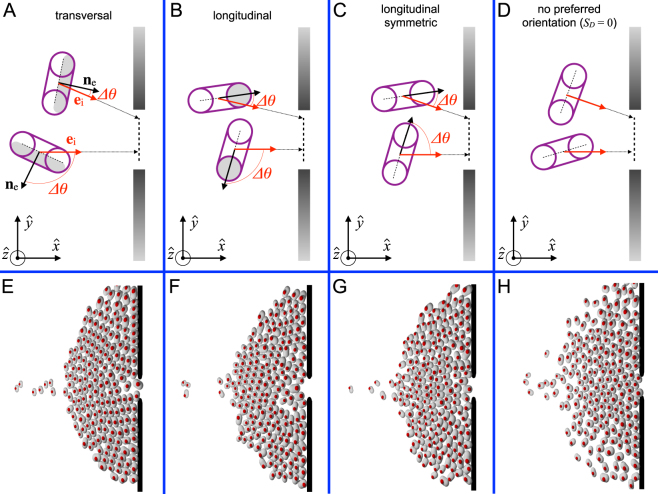
Sketches of the different type of particles’s orientation implemented in this work (**A**–**D**) and pictures of the emerging collective configurations (**E**–**H**). The direction of the desired velocity is characterized by a unitary vector **e**_*i*_ (red arrows in **A**) that points to the closest point over a segment 20 cm shorter than the door (dark dashed lines in **A**–**D**). The orientations at which each particle aims to move are defined by an unitary vector (**n**_*e*_) shown as a dark arrow. As the desired orientation of the particles coincides with the direction of **e**_*i*_, all particles with preferred orientation (**A**–**C**) apply a torque that is proportional to the difference among these two unitary vectors, Δ*θ*.

This *desired torque* has a linear part proportional to Δ*θ* ∈ [−*π*, *π*], the angular difference between the particle orientation and the desired one (Fig. 1). The parameter *S*_*D*_ that multiplies Δ*θ* can be seen as the angular strength. The desired torque also contains a damping force, which is $\beta =4.5\sqrt{{S}_{D}}$ times the angular velocity $\dot{\theta }$, guaranteeing over-damped conditions. Finally, a sinusoidal noise ${\Re }(t)$ is introduced to mimic the extra torque exerted by particles whenever trying to escape from a jammed situation. Inspired by the pedestrian case^18^, this noise was given the form ${\Re }(t)=\eta \,sin(\omega \,t+{\varphi }_{i})$ and is characterized by the period $T=\frac{2\pi }{\omega }=1.0\,{\rm{s}}$ and the amplitude *η* = 0.75 *mgL*; where *g* = 10 m/s^2^ and *m* and *L* are the largest particles’ mass and long axis, respectively.

**Figure 2:**
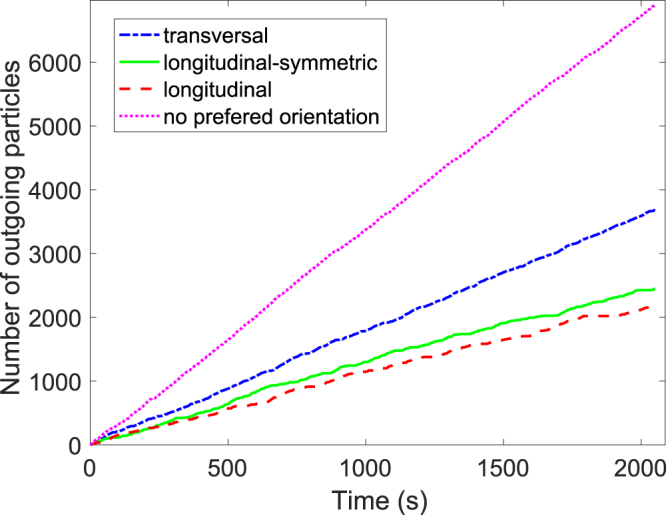
Number of particles that have left the room versus time for *W* = 0.7 m, *v*_*d*_ = 2.0 m/s, *S*_*D*_ = 15 Nm and three different preferred orientations as indicated in the legend. Also, the special case of *S*_*D*_ = 0 (no preferred orientation) is displayed for comparison.

A central concept of the present model is to define the particle orientation with respect to the moving direction, which is different for each type of particle investigated. We define it with a unitary vector **n**_*e*_ (dark arrows in Fig. 1A–C) that takes the following values: (1) in *transversal orientation*, **n**_*e*_ is perpendicular to the particle’s long axis and has a predefined sense (from the particle center to the grey side in Fig. 1A); (2) in *longitudinal (asymmetric) orientation*, **n**_*e*_ is aligned to the particle’s long axis and has a predefined sense (from the particle center to the grey side in Fig. 1B); and (3) in *longitudinal-symmetric orientation*, *n*_*e*_ is aligned to the particle’s long axis and has not a predefined sense (Fig. 1C); in this case, as the ‘head’ of the particle is (by definition) the end closer to the exit, Δ*θ* ∈ [−*π*/2, *π*/2]. Finally, a special case of no desired orientation is considered, i.e. *S*_*D*_ = 0.

Considering the definition of particle orientation introduced above, the desired orientation of each particle coincides with the direction of the target (red-ligth arrows in Fig. 1A–D); i.e. particles aim to get **n**_*e*_ aligned with **e**_*i*_. Incidentally, note that *longitudinal (asymmetric) propulsion* is analogous to the propulsion of sheep^12^ or janus rods^28^, and transversal propulsion corresponds to the case of humans.

In our simulations, the translational (Eq. 1) and rotational (Eq. 2) equations of motion of each particle were integrated using velocity Verlet and leap-frog algorithms, respectively. In particular, we adopted a hybrid CPU-GPU discrete element algorithm which benefits from the highly parallel structure of the NVIDA graphics processing units (GPUs). The algorithm has already been successfully used in the simulation of non-circular pedestrians^18^ and inert grains with different shapes^33–35^.

## Simulated Scenarios

The geometrical configuration of the system is the same than in^18^: a square domain of 8 × 8 m with a door of width *W* at the center of one of the sides. In all simulations 192 spherocylinders are implemented having the long axis *d*_*l*_ distributed in the range [0.35–0.5 m] and the short axis *d*_*s*_ in [0.24–0.33 m]. The simulated particles have an average mass of 67 kg with a normal distribution truncated at [45–114 kg]. All these parameters correspond to the real pedestrian experiments reported in^14^ but similar behavior is expected for other particle sizes as soon as the exit/particle size ratios are kept around the same values.

Initially, the self-propelled particles are homogeneously distributed within the enclosure at the vertices of a 12 × 16 square lattice (16 particles arranged in the direction perpendicular to the door) and randomly oriented. Then, they begin moving towards the exit, in such a way that the direction of the desired velocity **e**_*i*_ points to the closest point over a segment aligned with the door (and 20 cm shorter) as can be seen in Fig. 1A–D. In order to avoid transient effects^36^ related to the system’s initial state and guarantee stationary conditions we have implemented periodic boundary conditions^18^. In Fig. 1E–H we illustrate snapshots of typical configurations corresponding to the three kind of agents simulated and the case of no preferred orientation (*S*_*D*_ = 0). The simulations were run for *T*_*end*_ = 2000 s for each set of parameters. During this time, the number of circulating particles range from ~1500 to ~7500, depending on the conditions.

The parameters studied were the particle orientation (3 different options), door width *W*, desired velocity *v*_*d*_, and the angular strength *S*_*D*_. Based on our previous knowledge, the three parameters took values of: *W* = {0.6, 0.7, 0.8, 0.9, 1} m; *v*_*d*_ = {0.5, 1, 1.5, 2, 2.5, 3} m/s; and *S*_*D*_ = {0, 2, 5, 10, 15, 20} Nm.

## Results

### Flow rates

We start by presenting the number of particles that flow out of the enclosure versus time, when the different types of particle orientation are implemented keeping constant the rest of parameters (Fig. 2). Clearly, the best scenario possible is when particles propel without any preferred orientation. We also observe that the flow for transversal propulsion is considerably higher than for the longitudinal cases (which are similar each other). This result is rather surprising as the number of particles that are necessary to span the whole outlet is notably smaller if they propel transversally than when propel longitudinally (around 1.65 against 2.46). In what follows, we will show that this counter-intuitive behavior is robust and happens for all the directional strengths, desired velocities, and outlet sizes.

To this end, we look at the global specific flow rate (*Q*_*s*_) during the whole simulation run which can be computed as ${Q}_{s}=\frac{{N}_{tot}-{N}_{150}}{({T}_{end}-{T}_{150})\times W}$, where *N*_*tot*_ is the total number of pedestrians that cross the door in the time up to *T*_*end*_ = 2000s and the sub-index 150 indicates that we discard the first 150 outgoing particles to avoid the initial transient^18^.

**Figure 3:**
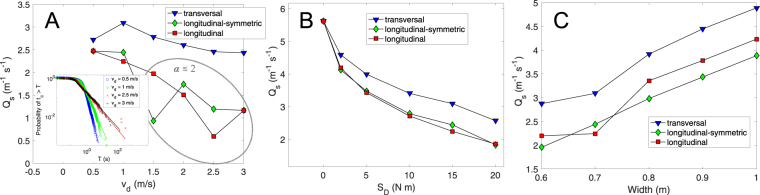
(**A**) Global flow rate versus desired velocity for *W* = 0.7 m, *S*_*D*_ = 15 Nm and three different orientations as indicated in the legend. The inset shows the survival function for the longitudinal case and four desired velocities (the two lowest and the two largest). (**B**) Global flow rate versus rotational strength for *W* = 0.7 m, *v*_*d*_ = 1 m/s and three different orientations as indicated in the legend. (**C**) Global flow rate versus door size for *v*_*d*_ = 1 m/s, *S*_*D*_ = 15 Nm and three different orientations as indicated in the legend.

Figure 3A displays the global specific flow rate for *W* = 0.7 m and *S*_*D*_ = 15 Nm versus the desired velocity for the three kind of orientations analyzed. In all cases, the transversal orientation gives rise to the higher flow rates whereas the two longitudinal orientations (symmetric and asymmetric) lead to similar outcomes. This confirms that, despite transversal orientation implies a larger cross section of the particle’s size with respect to the exit, the flow rate is notably larger. Apart from this, we should also stress that the faster-is-slower effect^37,38^ is present in all cases, hence demonstrating that this effect is robust and does not depend on the orientation at which particles propel. Finally, note the significant flow rate fluctuations obtained for desired velocities ${v}_{d}\gtrsim 1.5\,{\rm{m}}/{\rm{s}}$ in the case of longitudinally propelled particles. The origin of this is the development of very long clogs which strongly determine the global flow rate. Indeed, for several systems of frictional particles flowing through bottlenecks, it was already reported that the distributions of clog duration (*t*_*c*_) follow a power-law^1^. For the cases indicated in Fig. 3A we calculated the power-law exponents using the method proposed by Clauset *et al*.^39^ which are shown in Table 1. Values of $\alpha \lesssim 2$ imply that the flow rates obtained depend on the duration of the observation window, approaching zero for very long observation times.

**Table 1:** Power-law exponents (*α*) for the the longitudinal propelled cases shown in Fig. 3A.

*α* exponents	*vd* = 0.5 m/s	*vd* = 1.0 m/s	*vd* = 1.5 m/s	*vd* = 2.0 m/s	*vd* = 2.5 m/s	*vd* = 3.0 m/s
longitudinal	4.24 ± 0.26	2.81 ± 0.15	2.27 ± 0.04	2.06 ± 0.04	1.95 ± 0.09	1.89 ± 0.05
long. symmetric	3.99 ± 0.16	2.76 ± 0.09	2.22 ± 0.07	2.07 ± 0.04	1.98 ± 0.04	1.94 ± 0.08

In Fig. 2B we show the average specific flow rates for *W* = 0.7 m and *v*_*d*_ = 1 m/s versus the directional strength. Again, the transversal orientation reveals higher flow rates than the two longitudinal ones. The later are very similar, hence confirming that the preference of sense has little effect on the flow rate. In addition, we observe that in all cases the flow rate reduces monotonously with *S*_*D*_. This means that the case with no preferred orientation (*S*_*D*_ = 0) is the one where the flow rate through bottlenecks is maximized. Importantly, these results contradict the idea suggested in^18^ where the reduction of flow rate when increasing *S*_*D*_ in pedestrian like particles (transversally propelled) was attributed to the increase of the cross section in the direction parallel to the outlet. And this is so because the flow rate also reduces when increasing *S*_*D*_ in longitudinally oriented particles, which reduce the effective cross section as they become more aligned.

Finally, we report the outcomes of the flow rate when varying the exit size and keeping the rest of the parameters constant (Fig. 3C). Once more, the flow rate attained when the particles tend to propel transversally is the highest; definitively proving the robustness and generality of this behavior. Moreover, Fig. 3C reveals a weak difference in the flow rates reached by both longitudinally propelling particles: the symmetric ones, which have no preference of sense, shows up lower flow rates, at least for the largest exit sizes.

Up to now, we have reported several features of the influence of particle orientation with respect to moving direction on the flow rate through bottlenecks that can be summarized as:

- Particles with transversal orientation give rise to higher flow rates than longitudinal ones, despite the higher cross section of the former with respect to the exit direction.
- Particles that move with no preferred orientation reveal the highest flow rates.
- For large exits, longitudinally propelled particles evidence slightly higher flow rates when they have a predefined sense of movement.

In what follows we will justify all these features based on a very simple idea: the flow is highly conditioned by the formation of clogs at the outlet. Therefore, the stability of clogging configurations against the perturbations that might destroy them (which come mainly from the noise in the desired torque) is crucial in determining the flow rate. And, clearly, the particles orientation in the ensembles that are clogged must play a role in their stability.

### Desired vs. achieved orientation

**Figure 4:**
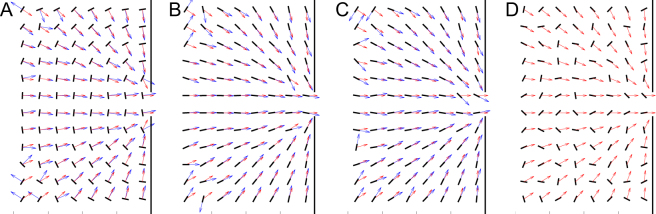
The most probable alignment of the particles analysed over a squared grid of 50 × 50 cm^2^. Black lines represent the most probable alignment of particles’ long axis, and blue arrows the corresponding definition of orientations (${{\bf{n}}}_{e}$). (**A**) Transversal; (**B**) longitudinal; (**C**) longitudinal symmetric; and (**D**) no preferred orientation. For reference, red arrows indicate the exit direction ${{\bf{e}}}_{i}$ which for cases (**A**–**C**) coincides with the particles’ desired orientation. The simulation conditions implemented to obtain these maps were: *W* = 0.7 m, *v*_*d*_ = 2 m/s and *S*_*D*_ = 20 Nm.

First of all, we present a general overview of the particles orientation in the room displaying the more probable alignment for each kind of agent in a grid of 50 × 50 cm^2^ (Fig. 4).

The outcomes confirm what one can expect from the prescription given in the model sketched in Fig. 1: longitudinally propelled particles align with their long axis pointing the exit, transversally propelled ones align perpendicularly to the exit direction, and particles without preferred orientation seem to be randomly oriented within the room. Having said that, it is remarkable that the deviation of the particle’s alignment with respect to the desired alignment (difference between blue and red vectors) seems slightly stronger for the case of transversal orientation than longitudinal. This effect can be explained considering that in a system of anisotropic particles with velocity gradients (as it is the bottleneck flow) shear forces lead to the emergence of torques that align particles with their long axis in the flow direction; i.e. in a way similar to the longitudinal case. This feature was already observed when studying the flow of elongated inert particles out of a silo under the action of gravity^22–25^. Therefore, for longitudinal orientation the torque resulting from shear forces acts on the same direction than the desired torque, aligning particles with the long axis pointing towards the exit. Oppositely, for transversal orientation these two torques act in different directions leading to what can be seen as a source of noise.

**Figure 5:**
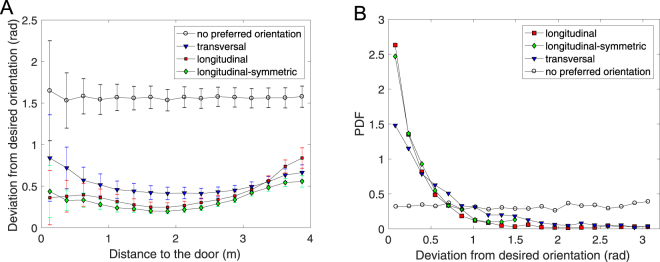
(**A**) Average of the absolute value of the deviation between actual and desired orientation as a function of the distance to the center of the door measured in sections of *δr*  =  0.25 m for *S*_*D*_ = 20 Nm, *v*_*d*_ = 1 m/s, *W* = 0.7 m and for particles propelling with different orientations with respect to the moving direction as indicated in the legend. (**B**) Distribution of the deviations between actual and desired orientations for particles at a distance of the door smaller than 0.75 m and the same conditions than (a).

Aiming a better quantification of this behavior, in Fig. 5 we analyse the difference between the actual and desired orientation (Δ*θ*) of the particles.

First, we display the average of the absolute value of this difference 〈|Δ*θ*|〉 as a function of the distance to the door (Fig. 5A). Note that for the case of no preferred orientation we take as a reference the orientation that would correspond to the longitudinal alignment. Clearly, the obtained values for this specific case (around *π*/2) indicate that the particles take any possible orientation (|Δ*θ*| ∈ [0, *π*]) with the same probability; hence revealing that the actual alignment by shear forces is very weak. When comparing the outcomes for the three different orientations we confirm that the deviation from desired orientation is higher for transversal propulsion. This feature becomes more obvious near the door, where the shearing forces are expected to be higher. The cases of symmetric and non-symmetric longitudinal orientation are indistinguishable.

Now, in order to look with more detail at the region near the door, we present the distribution of orientation deviations of those particles being at a distance to the exit smaller than 0.75 m in Fig. 5B. As expected, the distribution for longitudinal orientation is much narrower than the one for transversal case. This result confirms that the longitudinal propelled particles align to each other rather easily, hence giving rise to highly ordered structures that favor clogging. The alignment seems attenuated when particles propel transversally, so the flow is higher in this scenario. The limit case of particles without preferred orientation is the one with more disorder and therefore the best alternative to prevent clogging in constrictions.

**Figure 6:**
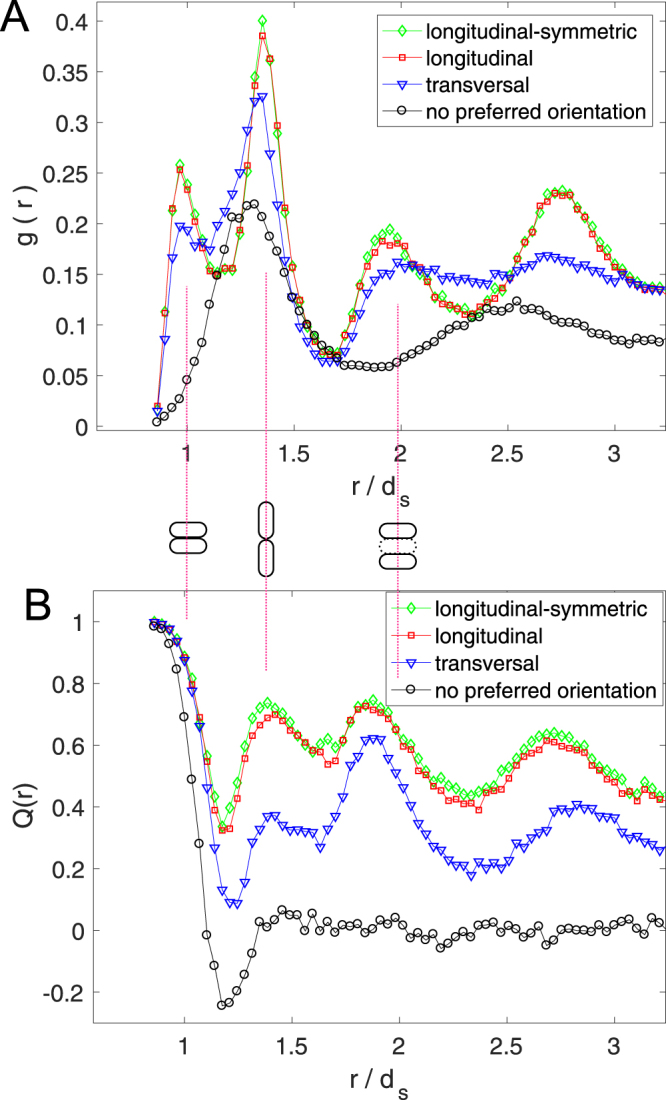
(**A**) g(r) and (**B**) Q(r) of particles laying in the area between 0 and 2 m from the exit when flowing out of the *W* = 0.7 m bottleneck with *v*_*d*_ = 1.0 m/s and *S*_*D*_ = 15 Nm for three different propulsion orientations as indicated in the legend. Also, the special case of *S*_*D*_ = 0 (no preferred orientation) is displayed for comparison. Note that the distance (*r*) has been rescaled by the particle’s short diameter (*d*_*s*_).

To confirm these ideas we have calculated the radial distribution function *g*(*r*) and the orientation distribution function *Q*(*r*) of the whole ensemble of particles for the different cases (Fig. 6).

The *g*(*r*) accounts for the probability of finding any two particles whose centers are at a distance *r*. It evidences peaks at distances that coincide with the particle short (*d*_*s*_) and long diameters and the sum of these; i.e. *r*/*d*_*s*_ = 1, 1.4, 2, 2.8... m. These peaks are a signature of very dense packings. Indeed, the fact that for the particles without any preference of orientation, most of the *g*(*r*) structure disappears correlates with the high degree of disorder deduced from the distributions of Fig. 3B. Following this line of reasoning, the *g*(*r*) outcomes also support that transversally oriented moving particles lead to higher disorder; as the height of the peaks in this case is distinctly lower than for longitudinally propelled agents. Apart from the ordering, we quantify particle’s alignment by means of the *Q*(*r*) which stands for the probability that two particles are aligned to each other, given that their centers are at a distance *r*. The specific equation for *Q*(*r*) can be found in^11^ but the idea is that when two particles are perfectly aligned *Q* = 1, whereas when their angles differ in *π*/2, then *Q* = −1. The obtained distributions definitively prove that the alignment developed when particles propel longitudinally is much higher than in the transversal case. Also, the values of *Q* ≈ 0 reached for *r*/*d*_*s*_ > 1.4 for the case of particles without preferred propulsion orientation, confirm the complete absence of order in this case. Incidentally, note that the peaks in the *Q*(*r*) appear for values of *r*/*d*_*s*_ slightly smaller than the ones at which were displayed in the *g*(*r*); an issue which is attributed to the polydispersity of the particles in terms of the size of their short and long diameters.

## Discussion

In this work we investigate the influence of the orientation at which asymmetric active particles propel on their flow through a constriction. First, we show that all kind of self-orientation mechanisms lead to the well known faster-is-slower effect evidencing its robustness against this property. In a similar way, increasing the angular strength (*S*_*D*_) implies a reduction of flow rate in all cases. This result is understood if we are aware that the clogging structures that interrupt the flow can be only destroyed by means of the angular noise intrinsic to each particle. As the magnitude of this noise has a constant value, its effect (in terms of ability of breaking arches) reduces when the angular strength increases.

Concerning the features associated to the different propulsion orientations, we discover that the best solution to maximize the outflow through a bottleneck is that particles propel without any preferred orientation. Probably due to the intrinsic noise existing in the desired torque, this strategy leads to highly disordered structures near the outlet, hence reducing clogging. The same reasoning applies to understand why longitudinally propelled particles lead to lower flow rates than transversally propelled ones (despite the former have a smaller cross section in the direction of the exit). Clearly, transversally propelled particles give rise to less ordered structures where particles are not well aligned to each other, an effect attributed to shear forces that give rise to torques acting in a different direction than the one desired by the particles.

Finally, let us focus on the small effect that the propulsion sense of longitudinally propelled particles has in the outflow. In principle, it sounds reasonable that both symmetric and asymmetric particles give rise to similar outcomes. Certainly, if asymmetric particles point towards the exit with their desired sense, the global configurations reached in both cases are equivalent. The only difference among these two kind of agents might arise if, for some reason, collective forces align an asymmetric particle in the direction of the door, but pointing backwards. In that case, the efforts of such misaligned particle to rotate 180 degrees, will introduce a local disorder in the system that would surely provoke a slight improvement of the flowability. As indicated by Fig. 3C, this phenomenon is more evident for large exits, suggesting that those are necessary in order to get sufficiently high shearing and disorder within the sample to completely misalign a longitudinally propelled particle.

In this manuscript we have examined what it could be considered the simplest example of anisotropic particles that propel along three basic orientations. Despite this simplicity, we believe that our results could set the basis to understand the flow of active particles through constrictions, whether real ones or artificially designed. More importantly, our findings can be straightforwardly connected to recent works about bottleneck flow of pedestrians, sheep or mice. Indeed, the smaller variation of the flow rate with desired velocity observed for transversal orientation than for longitudinal one (Fig. 3A) is perfectly compatible with the outcomes reported in^38^; where the variation of flow rates when changing competitiveness were significantly lower for pedestrians (around 7%) than sheep (around 19%).
